# Functions and regulation of lipocalin-2 in gut-origin sepsis: a narrative review

**DOI:** 10.1186/s13054-019-2550-2

**Published:** 2019-08-02

**Authors:** Fanglin Lu, Kei Inoue, Jungo Kato, Shizuka Minamishima, Hiroshi Morisaki

**Affiliations:** 1 Keio University Graduate School of Medicine Doctoral Programs, Tokyo, Japan; 2 Department of Anesthesiology, Keio University School of Medicine, 35 Shinanoamchi, Shinjuku-ku, Tokyo, 160-8582 Japan

**Keywords:** Sepsis, Lipocalin-2, Gastrointestinal microbiome, Inflammation, Neutrophils, Macrophages

## Abstract

Lipocalin-2 (Lcn2), an innate immune protein, has come to be recognized for its roles in iron homeostasis, infection, and inflammation. In this narrative review, we provide a comprehensive description based on currently available evidence of the clinical implications of Lcn2 and its therapeutic potency in gut-origin sepsis. Lcn2 appears to mitigate gut barrier injury via maintaining homeostasis of the microbiota and exerting antioxidant strategy, as well as by deactivating macrophages and inducing immune cell apoptosis to terminate systemic hyper-inflammation. We propose that development of a therapeutic strategy targeting lipocalin-2 could be highly promising in the management of gut-origin sepsis*.*

## Background

Despite the numerous advances in the field of critical care, sepsis remains a widely recognized health concern causing or contributing to up to 5.3 million deaths on a worldwide scale per annum [1]. Indeed, the World Health Assembly and World Health Organization made sepsis a global health priority in 2017 by adopting a resolution to improve, prevent, diagnose, and manage sepsis. Sepsis has recently been re-defined as a life-threatening organ dysfunction caused by a dysregulated host response to infection [2]. While the latest Surviving Sepsis Campaign guidelines 2016 provide a number of evidence-based recommendations on the management of patients with sepsis [3], significant knowledge gaps remain on a wide array of issues, including in relation to the fundamental mechanisms underlying the pathogenesis of sepsis, the potential of tailored medicine approaches for sepsis, and monitoring of the response to treatment [4].

As the Hippocratic quote goes “All disease begins in the gut”, the concept of the gut being the motor of systemic inflammatory response syndrome (SIRS) and multiple organ dysfunction syndrome (MODS) in critically ill patients is referred to as gut-origin sepsis [5, 6]. Conversely, extra gut-origin sepsis such as *Pseudomonas aeruginosa* pneumonia is also known to enhance epithelial apoptosis and decrease intestinal villi, resulting in gut barrier dysfunction [7]. In other words, the gut injury becomes sometimes the consequence instead of the origin of sepsis. According to the first hypothesis of gut-origin sepsis, overgrowth of pathogenic microbiome, termed “dysbiosis,” and intestinal barrier failure are involved in the development of SIRS and MODS through translocation of enteric bacteria and/or pathogen-associated molecular patterns (PAMPs), such as lipopolysaccharide (LPS), from the intestinal lumen to the portal vein in patients with sepsis [8–10]. This initial hypothesis was, however, not fully supported by clinical data; studies revealed no microbes or their products in the portal blood in patients with severe trauma [11].

**Fig. 1:**
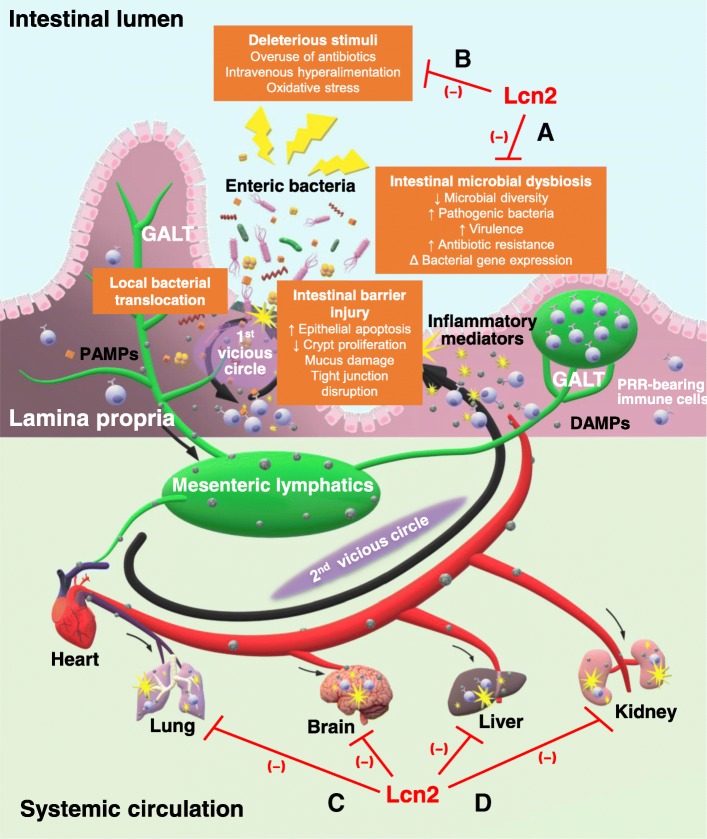
Gut-lymph hypothesis of gut-origin sepsis and functions of lipocalin (Lcn) 2. Various deleterious stimuli can induce intestinal barrier injury and microbial dysbiosis. The invasion by translocated enteric bacteria or their PAMPs and gut-derived DAMPs sets off a local inflammatory response by immune cells stored in GALT, further impairing the intestinal barrier (first vicious circle). Mesenteric lymphatics transport DAMPs to the systemic circulation where their recognition by immune cells triggers the development of SIRS and MODS (second vicious circle). Here, the therapeutic potential of Lcn2 is considered. Inside the gut, Lcn2 is suggested to maintain intestinal microbiota homeostasis (a) and protect the intestinal barrier against oxidative stress (b). When systemic hyper-inflammation occurs, Lcn2 bolsters its termination (c) and shield organs from MODS (d). *GALT* gut-associated lymphoid tissue, *DAMPs* danger-associated molecular patterns, *PAMPs* pathogen-associated molecular patterns, *PRR* pattern recognition receptor, *SIRS* systemic inflammatory response syndrome, *MODS* multiple organ dysfunction syndrome

Soon thereafter, the role of the gut-associated lymphoid tissue (GALT), the largest immune organ in the body, entered the spotlight. Mesenteric lymphatics, a major part of GALT, which drain into the pulmonary circulation, were suggested as the new pathway connecting gut barrier dysfunction with the development of MODS. A series of elaborate studies in Deitch’s laboratory provided formidable evidence for the gut-lymph theory, according to which injured gut mucosa excretes danger-associated molecular patterns (DAMPs) into the mesenteric lymphatics, which then enter the systemic circulation, irrespective of bacterial translocation, via the portal vein [12]. These injurious biomolecules are then recognized by pattern recognition receptor (PRR)-bearing innate immune cells (e.g., macrophages, leukocytes, and dendritic cells), triggering the onset of SIRS and MODS, including acute lung injury or acute respiratory distress syndrome, and deteriorative gut failure (Fig. 1).

Lipocalin-2 (Lcn2), also known as neutrophil gelatinase-associated lipocalin (NGAL), oncogene 24p3, or siderocalin, is an innate immune protein that was first discovered from a simian virus 40-infected murine kidney cell culture [13], and its human protein analog NGAL was later found to be associated with matrix metalloproteinase (MMP)-9, a gelatinase secreted by neutrophils for extracellular matrix remodeling [14, 15]. For many years, Lcn2 has been recognized as an emerging player in different physiological and pathological processes, including iron homeostasis, inflammation, microbial infection, organogenesis, neurodegeneration, and tumorigenesis [16–18]. Meanwhile, its broad expression in various tissues and cell types and its detectable size and relative stability make it potentially useful both as a diagnostic biomarker and as a prognostic indicator for several diseases, including inflammatory bowel disease [19, 20] and sepsis-related acute kidney injury [21, 22]. In the field of intestinal inflammation and critical illnesses, Lcn2 appears to be of clinical relevance in gut-origin sepsis.

The aim of the present review is to provide new insights into the possible roles of Lcn2 in gut-origin sepsis and to pave the way for developing treatment strategies targeting Lcn2 for sepsis.

## Expression and modulation of Lcn2 in gut-origin sepsis

A decade ago, the production and expression of Lcn2 in the intestinal epithelium was demonstrated in a rhesus macaque model of bacterial infection [23] and by administration of indomethacin in mice [24]. Using a murine model of LPS-induced endotoxemia, we further confirmed the presence of Lcn2 in the intestinal crypt cells under physiological conditions and demonstrated the existence of correlations between the severity of illness in critically ill individuals and the accumulation of Lcn2 in the crypt lamina of the ileum and colon and its discharge into the intestinal lumen, the first scene of gut-origin sepsis [25].

Elevated levels of Lcn2 protein in the plasma after bacterial infection has been described in animal models as well as in critically ill patients, indicating the potential functions and/or roles of Lcn2 in the pathogenesis of sepsis [21, 22, 26]. Elevated expression levels of both Lcn2 gene and protein in the hepatocytes, and release of Lcn2 protein into the systemic circulation, were detected in a mouse model of sepsis [27–30]. Xu et al. also strongly contended that hepatocytes are responsible for more than 90% of the elevated circulating Lcn2 levels, which relies on the hepatic signal transducers and activator of transcription (STAT) 3 signaling pathway activated by interleukin (IL)-6 [31].

Apart from the intestinal epithelial cells and hepatocytes, innate immune cells such as neutrophils and macrophages have also been shown in in vivo experiments to secrete Lcn2 after LPS administration, via activation of the toll-like receptor (TLR)-4, one of the PRRs [27–30, 32, 33]. In addition to LPS, which has been identified as the major stimulator of Lcn2 expression in the aforementioned cell types, induction of Lcn2 can also be triggered by other TLR ligands [25, 34–37] and cytokines, including IL-1β [38], IL-6 [31, 39], IL-10 [30, 40, 41], IL-17 [23, 38, 42], IL-22 [23, 42], and tumor necrosis factor-α [42], depending on the activation of the nuclear factor-kappa B (NF-κB)-STAT3 loop (Table 1). Collectively, inducible intra- and extra-gut expressions of Lcn2 by infection- and inflammation-related molecules suggest that Lcn2 plays important roles in gut-origin sepsis.

**Table 1:** Modulation and expression of lipocalin-2 during gut-origin sepsis

Inducers		Lcn2	Cell types	References
TLR ligands	Zymosan	↑	Intestinal epithelial cells	[25]
	PGN, LTA	↑	Macrophages	[34]
	Poly I: C	↑	Intestinal epithelial cells Microglia	[35–37]
	LPS	↑	Intestinal epithelial cells Hepatocytes Macrophages Blood cells and peritoneal cells	[25, 27–30, 32, 33, 38]
	Flagellin	↑	Intestinal epithelial cells	[35]
	CpG-DNA	↑	Intestinal epithelial cells	[25]
Cytokines	IL-1β	↑	Intestinal epithelial cells	[38]
	IL-3	**↓**	Hematopoietic cells	[43]
	IL-6	↑	Hepatocytes Macrophages	[31, 39]
	IL-10	↑	Macrophages	[30, 40, 41]
	IL-17A	↑	Intestinal epithelial cells	[23, 38, 42]
	IL-17+IL-22	↑	Intestinal epithelial cells	[23, 42]
	IL-17+TNF-α	↑	Intestinal epithelial cells	[42]

*TLR* toll-like receptor, *PGN* peptidoglycan, *LTA* lipoteichoic acid, *Poly I: C* polyinosinic-polycytidylic acid, *LPS* lipopolysaccharide, *NF-κB* nuclear factor-kappa B, *STAT3* signal transducer and activator of transcription 3, *TNF-α* tumor necrosis factor-alpha

## Intra-gut functions of Lcn2 in gut-origin sepsis

As depicted in Fig. 1, bacterial translocation and local immune responses trigger a vicious intra-gut circle of intestinal barrier injury and downstream events, and followed by another vicious circle in systemic circulation to develop MODS. Two major therapeutic approaches to break these vicious circles in the early phase are (a) restoration of the microbiota homeostasis, for example, selective digestive tract decontamination and use of probiotics, prebiotics, and synbiotics, and (b) prevention of intestinal barrier injury via early resuscitation, enteral nutrition, immunonutrition, and administration of antioxidants [9, 12]. Since the safety and effectiveness of these clinically available therapies are yet to be established, there is an emerging need for new therapeutic options in these directions. Accumulating evidence suggested the potential usefulness of Lcn2 in this regard.

### Microbiota homeostasis

Singh et al. demonstrated significantly reduced serum and fecal Lcn2 levels in germ-free mice and that the levels could be increased by oral gavage of cecal contents from wild-type mice. In addition, Lcn2-null mice exhibited gut bacterial dysbiosis with an increase in the bacterial burden and proportion of gram-negative bacteria. In other words, Lcn2, being microbiota-inducible by itself, could be a prerequisite to maintain microbiota homeostasis [35].

#### Extracellular bacteriostasis

According to available evidence, iron is essential for life and is stringently maintained at a low concentration by iron-binding proteins (e.g., ferritin, transferrin) [44]. Notwithstanding the scarcity of available iron, bacteria manage to synthesize siderophores to scavenge iron from the host proteins and transport it back to themselves [45]. In 2004, an in vitro growth assay performed by Flo et al. elegantly demonstrated that exogenous Lcn2 directly inhibited the growth of *Escherichia coli* (*E. coli*) via its binding specificity for catecholate-type siderophores such as enterochelin, as suggested by its alias, siderocalin. This direct dose-dependent extracellular bacteriostatic function of Lcn2 against *E. coli*, one of the major pathogens of gut-origin sepsis, has also been shown by other groups in in vitro experiments [25, 46–48].

#### Bacteriostasis by macrophages

Intestinal macrophages in the lamina propria are considered to function as a firewall against bacteria that have translocated across the mucosal barrier via phagocytosis [49]. As these macrophages are derived from blood monocytes, they do not express any innate response receptors against LPS or produce any inflammatory cytokines. Thus, they retain avid phagocytic and bactericidal activity without initiating overt inflammation [50]. In a study of peritoneal macrophages obtained from Lcn2-null mice cultured with *E. coli*, Toyonaga et al. demonstrated weakened intracellular bacterial clearance by macrophages as compared to the macrophages from wild-type mice, which could be reversed by administration of recombinant Lcn2 [38]. Similar restriction of intramacrophage bacterial growth by limiting iron availability to the pathogens was also shown in macrophages infected by *Salmonella typhimurium* [32, 51, 52], *Chlamydia pneumoniae* [53], and *Mycobacterium tuberculosis* [47]. However, it should be noted that the macrophages employed in the aforementioned experiments were not intestinal, but bone marrow-derived or peritoneal macrophages. The results therefore need to be interpreted with caution.

#### Bacteriostasis by neutrophils

Neutrophils serve as the first line of the host defense system against bacterial infections, that is, they migrate to the site of infection and kill the bacteria phagocytosed by them. Being named as a neutrophil protein, Lcn2 can be considered as necessary to ensure normal functioning of neutrophils. In one in vitro experiment, impaired activation of genes critical for mobility (e.g., RhoA) despite chemoattractant stimulation was reported in neutrophils isolated from Lcn2-null mice [54]. Another study using mouse models of acute mycobacterial infection showed that Lcn2 accelerates macrophage production of the keratinocyte chemoattractant (KC/CXCL1) for neutrophil recruitment [55]. Of interest, Schroll et al. noted that polymorphonuclear neutrophils (PMNs) from Lcn2-null mice reacted with a delayed migratory potency and reduced expressions of adhesion molecules, including CD51 and CD11b, in response to intravenous LPS challenge. In addition, Lcn2 per se has been shown to attract murine PMNs in vivo and human PMNs in vitro, which might be related to Erk1/2-mediated signaling [56]. Nonetheless, contrary findings have also been reported, in that Lcn2 exerted no influence neutrophil recruitment and activation in mice at 24 h post-infection with *Klebsiella pneumoniae*, which could be attributable to differences in the models and analytical methods used among different studies [28, 57]. Taken together, it appears that Lcn2 might facilitate neutrophil recruitment in a paracrine as well as autocrine manner, because it is secreted by epithelial cells and neutrophils in response to certain inflammatory stimuli.

Once they arrive at the site of infection from the peripheral blood, neutrophils start to perform their antimicrobial functions (which can be suppressed by *E. coli*-derived enterobactin [58]), including phagocytosis (ingestion), degranulation (release of soluble antimicrobials such as myeloperoxidase), and formation of neutrophil extracellular traps (NETs) [59]. Peritoneal or bone marrow-derived neutrophils from Lcn2-null mice showed diminished phagocytotic activity when incubated with fluorescence-labeled *E. coli* [54], and furthermore, Lcn2 also rescued myeloperoxidase from enterochelin-mediated inhibition in vitro [60]. On the contrary, Li et al. argued that Lcn2 had no impact on neutrophil phagocytosis or degranulation ex vivo. They also contended that Lcn2 was not indispensable for NET formation, but contributed to the antibacterial functions of NETs in an iron-dependent manner [28]. This inconsistency, however, could also be attributable to differences in the models adopted.

To evade recognition by Lcn2, certain bacteria express Lcn2-resistant or stealth siderophores (e.g., salmochelin, aerobactin, yersiniabactin, and ferrichrome) [23, 27, 61–64], which requires to be taken into account when evaluating the practicality of applying Lcn2 in clinical situations. Notwithstanding, increased levels of Lcn2 were observed in patients with *Staphylococcus aureus* infection (bacterium whose siderophores are unrecognizable by Lcn2) or *Streptococcus pneumoniae* (bacterium that does not express any siderophores) [41]. These findings hint at multiple roles of Lcn2 other than as a siderophore sequestrant in the course of infection.

### Antioxidant effect

Since oxidative stress is also regarded as one of the major factors involved in the disruption of the intestinal barrier in gut-origin sepsis, a number of antioxidant therapies are now under trial [9]. Lcn2 has been demonstrated to exert protective effects against H_2_O_2_ toxicity in vitro [65, 66]. In addition, the presence or administration of Lcn2 induced hypoferremia of inflammation and upregulation of antioxidant enzymes (e.g., superoxide dismutase, heme oxygenase-1) in vivo, to limit iron-induced oxidative stress during sepsis [67, 68]. In other words, Lcn2 might protect against oxidative stress and aid in mitigating intestinal barrier injury. Clark and Coopersmith suggested that cumulative alterations and integrated perturbation in the crosstalk between intestinal epithelium, immune system, and commensal bacteria render the gut the “motor” of critical illness [69]. It should be noted that Lcn-2 could modulate independently each element of such intestinal crosstalk: bacteriostatic properties on disturbed gut microbiota, antioxidant effects on wounded gut barrier, and anti-inflammatory functions on runaway immune system described below can conduce to destroy this motor, thus breaking the vicious circles in gut-origin sepsis.

## Extra-gut functions in gut-origin sepsis

As Sir William Osler stated in his book, The Evolution of Modern Medicine, in 1904: “Except on few occasions, the patient appears to die from the body’s response to infection rather than from the infection,” our body enters a hyper-inflammatory stage when DAMPs released to circulation are recognized by immune cells in gut-origin sepsis, resulting in the development of multiple organ failure. Therefore, in addition to using antimicrobial strategies, it is crucial to quell the overwhelming inflammation and encourage tissue repair after the clearance of pathogens.

### Termination of the immune response

Over and above its primary role as a chelator of bacterial siderophores, Lcn2 is assumed as a modulator of inflammatory response, based on the findings of various studies. Lcn2 deficiency has been postulated to skew different types of macrophages (peritoneal or bone marrow-derived macrophages from mice or RAW264.7) toward M1 activation and to upregulate the expressions of pro-inflammatory cytokines (e.g., IL-1β, IL-6, and TNF-α) in response to LPS stimulation via activation of the NF-κB-STAT3 loop [38, 39, 67, 70, 71]. In addition, it was found that IL-10 potentiated LPS-mediated Lcn2 gene expression in macrophages [30] and that Lcn2 skewed the cells toward a deactivated phenotype via induction of IL-10 in mice with pneumococcal pneumonia [41]. Since the PAMPs shared by pathogens, of which LPS is a prototype, do not take part in the second vicious circle of gut-origin sepsis pursuant to the “gut-lymph” hypothesis, there is abundant room for more work to validate the roles of Lcn2 in the inflammatory response triggered by DAMPs (e.g., high-mobility group box 1 (HMGB1), IL-1 family), which stimulate PRRs, in a fashion similar to PAMPs [72–74].

On the other hand, preincubation with Lcn2 augmented staurosporine-induced cell death of macrophages by increasing caspase-3/7 activity [34], and Lcn2-null neutrophils failed to show granulocyte-colony-stimulating factor (G-CSF) deprivation-induced cell death [54]. Furthermore, Devireddy et al. revealed that Lcn2 can induce apoptosis in the absence upon the withdrawal of IL-3 [43, 75]. Iron-lacking Lcn2 is internalized by cells via the cell-surface receptor 24p3R on human and murine peripheral blood mononuclear cells, binds to a putative intracellular mammalian “siderophore” iron complex, and subsequently transports iron extracellularly via exocytosis. Consequently, intracellular iron depletion induces expression of the pro-apoptotic protein Bim, resulting in apoptosis [76]. When myeloid cells were cultured directly with Lcn2, however, no indication of an altered apoptotic or inflammatory state was detected [39, 77], underscoring the notion that Lcn2 per se may regulate neither inflammation nor apoptosis, but exerts anti-inflammatory and pro-apoptotic effects until a particular condition, as mentioned above, is met.

IL-3 is initially secreted by T cells to stimulate differentiation and proliferation of hematopoietic cells, and its secretion decreases as the immune response comes to an end [78]. It is thus rational that when the bacteriostatic role of immune cells is fulfilled and there are no more iron-binding siderophores to sequester, upregulation of Lcn2 by IL-3 withdrawal leads to macrophage deactivation as well as apoptotic death of those immune cells. Hence, although we are unable to assert that Lcn2 by itself has anti-inflammatory effects sufficient enough to generate the compensatory anti-inflammatory response syndrome (CARS) at this moment, Lcn2 may play a role as a negative regulator, contributing to resolution to excessive inflammation.

### Prevention of multiple organ failure

In addition to its major role in maintaining local microbiota homeostasis and moderating systemic overactive immune response, Lcn2 has also been reported to protect the gut as well as the lungs, liver, and kidneys from organ failure in sepsis models [28, 48, 67, 68]. Administration of recombinant Lcn2 in vitro to wounded monolayers of colonic epithelial cells promoted cell migration, and its subcutaneous administration in the rat model attenuated indomethacin-induced gastric injury in a dose-dependent manner [24]. Impaired/delayed liver regeneration in Lcn2-null mice after partial hepatectomy, Lcn2-inhibited renal apoptosis during peritonitis-induced sepsis, and Lcn2-enhanced reparative tubule formation in vitro have also been documented [31, 68, 79]. It is noteworthy that in spite of the contradiction to its foregoing pro-apoptotic effects on myeloid cells, the jury is still out since the apoptosis-related role of Lcn2 is seldom examined in the context of sepsis. Nevertheless, it remains to be clarified if Lcn2 that is secreted abundantly in the central nervous system following systemic LPS injection acts to protect or is deleterious to the central nervous system [17, 37]. Jang et al. showed downregulated mRNA expression of pro-inflammatory cytokines in Lcn2-null mice after intraperitoneal injection of LPS [80], while the opposite was shown by Kang et al. [81]. Further studies, taking these discrepancies into consideration, will need to be undertaken when exploring the systemic impact of Lcn2.

## Therapeutic potential

On the bench side, Lcn2-null mice manifested greater susceptibility to sepsis with a significantly accelerated mortality rate, higher bacterial load in organs, and more severe organ damages [27, 28, 31, 54, 64, 67], and Lcn2-null immune cells exhibited reduced local bactericidal activities and excessive systemic inflammatory responses [32, 38, 41, 47, 51–54]; in both of the above cases, the conditions could be rescued by exogenous administration of recombinant Lcn2. In view of all that has been discussed so far, it could be hypothesized that Lcn2 offers therapeutic promise against gut-origin sepsis through its game-changing abilities summarized in Fig. 1.

Disparities in the pathophysiologic effects of Lcn2 were observed depending on specific conditions, including the disease model, stage of illness, tissues/organs, and analytical methods employed, which might make the manipulation of this multifaceted protein during sepsis extremely tricky, because of the risk of causing the wrong war in the wrong place at the wrong time with the wrong enemy. For example, Warszawska et al. worried that elevation of the Lcn2 level by inhaled corticosteroids in critically ill patients with bacterial pneumonia might deactivate macrophages and in turn hamper effective bacterial clearance and aggravate the disease [41]. It is also important to bear in mind that the gut-lymph theory of gut-origin sepsis is merely one route plotted on the roadmap to understanding sepsis, and practical situations in the ICU can be exceedingly more complex and perilous than laboratory-controlled experiments.

## Conclusions

We have reviewed some characteristics of Lcn2, and much remains to be unveiled. Further research is warranted to comprehend the mechanism through which Lcn2 may harness the power of the host immune system to fight gut-origin sepsis, to provide bench side knowledge for bedside practice.

## Data Availability

Not applicable.

## Abbreviation

CARS: Compensatory anti-inflammatory response syndrome
DAMPs: Danger-associated molecular patterns
*E. coli*: *Escherichia coli*
GALT: Gut-associated lymphoid tissue
G-CSF: Granulocyte-colony-stimulating factor
HMGB1: High-mobility group box 1
IL: Interleukin
LPS: Lipopolysaccharide
LTA: Lipoteichoic acid
MMP-9: Matrix metalloproteinase-9
MODS: Multiple organ dysfunction syndrome
NETs: Neutrophil extracellular traps
NF-κB: Nuclear factor-kappa B
NGAL: Neutrophil gelatinase-associated lipocalin
PAMPs: Pathogen-associated molecular patterns
PGN: Peptidoglycan
PMNs: Polymorphonuclear neutrophils
Poly I: C: Polyinosinic-polycytidylic acid
PRR: Pattern recognition receptor
SIRS: Systemic inflammatory response syndrome
STAT3: Signal transducers and activator of transcription 3
TLR: Toll-like receptor
TNF-α: Tumor necrosis factor-alpha

## References

[1] Fleischmann C, Scherag A, Adhikari NK, Hartog CS, Tsaganos T, Schlattmann P (2016). Assessment of global incidence and mortality of hospital-treated sepsis. Current estimates and limitations. Am J Respir Crit Care Med.

[2] Singer M, Deutschman CS, Seymour CW, Shankar-Hari M, Annane D, Bauer M (2016). The Third International Consensus Definitions for Sepsis and Septic Shock (Sepsis-3). JAMA.

[3] Rhodes A, Evans LE, Alhazzani W, Levy MM, Antonelli M, Ferrer R (2017). Surviving Sepsis Campaign: International Guidelines for Management of Sepsis and Septic Shock: 2016. Intensive Care Med.

[4] Coopersmith CM, De Backer D, Deutschman CS, Ferrer R, Lat I, Machado FR (2018). Surviving sepsis campaign: research priorities for sepsis and septic shock. Intensive Care Med.

[5] Klingensmith NJ, Coopersmith CM (2016). The gut as the motor of multiple organ dysfunction in critical illness. Crit Care Clin.

[6] Otani S, Coopersmith CM (2019). Gut integrity in critical illness. J Intensive Care.

[7] Coopersmith CM, Stromberg PE, Davis CG, Dunne WM, Amiot DM, Karl IE (2003). Sepsis from Pseudomonas aeruginosa pneumonia decreases intestinal proliferation and induces gut epithelial cell cycle arrest. Crit Care Med.

[8] Fay KT, Ford ML, Coopersmith CM (2017). The intestinal microenvironment in sepsis. Biochim Biophys Acta Mol Basis Dis.

[9] Assimakopoulos SF, Triantos C, Thomopoulos K, Fligou F, Maroulis I, Marangos M (2018). Gut-origin sepsis in the critically ill patient: pathophysiology and treatment. Infection..

[10] Deitch EA (1990). Bacterial translocation of the gut flora. J Trauma.

[11] Moore FA, Moore EE, Poggetti R, McAnena OJ, Peterson VM, Abernathy CM (1991). Gut bacterial translocation via the portal vein: a clinical perspective with major torso trauma. J Trauma.

[12] Deitch EA (2012). Gut-origin sepsis: evolution of a concept. Surg.

[13] Hraba-Renevey S, Turler H, Kress M, Salomon C, Weil R (1989). SV40-induced expression of mouse gene 24p3 involves a post-transcriptional mechanism. Oncogene..

[14] Kjeldsen L, Johnsen AH, Sengelov H, Borregaard N (1993). Isolation and primary structure of NGAL, a novel protein associated with human neutrophil gelatinase. J Biol Chem.

[15] Triebel S, Blaser J, Reinke H, Tschesche H (1992). A 25 kDa alpha 2-microglobulin-related protein is a component of the 125 kDa form of human gelatinase. FEBS Lett.

[16] Chakraborty S, Kaur S, Guha S, Batra SK (2012). The multifaceted roles of neutrophil gelatinase associated lipocalin (NGAL) in inflammation and cancer. Biochim Biophys Acta.

[17] Ferreira AC, Da Mesquita S, Sousa JC, Correia-Neves M, Sousa N, Palha JA (2015). From the periphery to the brain: Lipocalin-2, a friend or foe?. Prog Neurobiol.

[18] Moschen AR, Adolph TE, Gerner RR, Wieser V, Tilg H (2017). Lipocalin-2: a master mediator of intestinal and metabolic inflammation. Trends Endocrinol Metab.

[19] Chassaing B, Srinivasan G, Delgado MA, Young AN, Gewirtz AT, Vijay-Kumar M (2012). Fecal lipocalin 2, a sensitive and broadly dynamic non-invasive biomarker for intestinal inflammation. PLoS One.

[20] Magro F, Lopes S, Coelho R, Cotter J, Dias de Castro F, Tavares de Sousa H (2017). Accuracy of faecal calprotectin and neutrophil gelatinase B-associated lipocalin in evaluating subclinical inflammation in UlceRaTIVE colitis-the ACERTIVE study. J Crohns Colitis.

[21] Bagshaw SM, Bennett M, Haase M, Haase-Fielitz A, Egi M, Morimatsu H (2010). Plasma and urine neutrophil gelatinase-associated lipocalin in septic versus non-septic acute kidney injury in critical illness. Intensive Care Med.

[22] Martensson J, Bell M, Oldner A, Xu S, Venge P, Martling CR (2010). Neutrophil gelatinase-associated lipocalin in adult septic patients with and without acute kidney injury. Intensive Care Med.

[23] Raffatellu M, George MD, Akiyama Y, Hornsby MJ, Nuccio SP, Paixao TA (2009). Lipocalin-2 resistance confers an advantage to Salmonella enterica serotype typhimurium for growth and survival in the inflamed intestine. Cell Host Microbe.

[24] Playford RJ, Belo A, Poulsom R, Fitzgerald AJ, Harris K, Pawluczyk I (2006). Effects of mouse and human lipocalin homologues 24p3/lcn2 and neutrophil gelatinase-associated lipocalin on gastrointestinal mucosal integrity and repair. Gastroenterology..

[25] Mori K, Suzuki T, Minamishima S, Igarashi T, Inoue K, Nishimura D (2016). Neutrophil gelatinase-associated lipocalin regulates gut microbiota of mice. J Gastroenterol Hepatol.

[26] Lentini P, de Cal M, Clementi A, D'Angelo A, Ronco C (2012). Sepsis and AKI in ICU patients: the role of plasma biomarkers. Crit Care Res Pract.

[27] Flo TH, Smith KD, Sato S, Rodriguez DJ, Holmes MA, Strong RK (2004). Lipocalin 2 mediates an innate immune response to bacterial infection by sequestrating iron. Nature..

[28] Li H, Feng D, Cai Y, Liu Y, Xu M, Xiang X (2018). Hepatocytes and neutrophils cooperatively suppress bacterial infection by differentially regulating lipocalin-2 and neutrophil extracellular traps. Hepatol.

[29] Sunil VR, Patel KJ, Nilsen-Hamilton M, Heck DE, Laskin JD, Laskin DL (2007). Acute endotoxemia is associated with upregulation of lipocalin 24p3/Lcn2 in lung and liver. Exp Mol Pathol.

[30] Vazquez DE, Nino DF, De Maio A, Cauvi DM (2015). Sustained expression of lipocalin-2 during polymicrobial sepsis. Innate immunity.

[31] Xu MJ, Feng D, Wu H, Wang H, Chan Y, Kolls J (2015). Liver is the major source of elevated serum lipocalin-2 levels after bacterial infection or partial hepatectomy: a critical role for IL-6/STAT3. Hepatol.

[32] Fritsche G, Nairz M, Libby SJ, Fang FC, Weiss G (2012). Slc11a1 (Nramp1) impairs growth of Salmonella enterica serovar typhimurium in macrophages via stimulation of lipocalin-2 expression. J Leukoc Biol.

[33] Meheus LA, Fransen LM, Raymackers JG, Blockx HA, Van Beeumen JJ, Van Bun SM (1993). Identification by microsequencing of lipopolysaccharide-induced proteins secreted by mouse macrophages. J Immunol.

[34] Eller K, Schroll A, Banas M, Kirsch AH, Huber JM, Nairz M (2013). Lipocalin-2 expressed in innate immune cells is an endogenous inhibitor of inflammation in murine nephrotoxic serum nephritis. PLoS One.

[35] Singh V, Yeoh BS, Chassaing B, Zhang B, Saha P, Xiao X (2016). Microbiota-inducible innate immune, siderophore binding protein lipocalin 2 is critical for intestinal homeostasis. Cell Mol Gastroenterol Hepatol.

[36] Ostvik AE, Granlund AV, Torp SH, Flatberg A, Beisvag V, Waldum HL (2013). Expression of toll-like receptor-3 is enhanced in active inflammatory bowel disease and mediates the excessive release of lipocalin 2. Clin Exp Immunol.

[37] Ip JP, Nocon AL, Hofer MJ, Lim SL, Muller M, Campbell IL (2011). Lipocalin 2 in the central nervous system host response to systemic lipopolysaccharide administration. J Neuroinflammation.

[38] Toyonaga T, Matsuura M, Mori K, Honzawa Y, Minami N, Yamada S (2016). Lipocalin 2 prevents intestinal inflammation by enhancing phagocytic bacterial clearance in macrophages. Sci Rep.

[39] Guo H, Jin D, Chen X (2014). Lipocalin 2 is a regulator of macrophage polarization and NF-kappaB/STAT3 pathway activation. Mol Endocrinol.

[40] Jung M, Weigert A, Tausendschon M, Mora J, Oren B, Sola A (2012). Interleukin-10-induced neutrophil gelatinase-associated lipocalin production in macrophages with consequences for tumor growth. Mol Cell Biol.

[41] Warszawska JM, Gawish R, Sharif O, Sigel S, Doninger B, Lakovits K (2013). Lipocalin 2 deactivates macrophages and worsens pneumococcal pneumonia outcomes. J Clin Invest.

[42] Stallhofer J, Friedrich M, Konrad-Zerna A, Wetzke M, Lohse P, Glas J (2015). Lipocalin-2 is a disease activity marker in inflammatory bowel disease regulated by IL-17A, IL-22, and TNF-alpha and modulated by IL23R genotype status. Inflamm Bowel Dis.

[43] Devireddy LR, Teodoro JG, Richard FA, Green MR (2001). Induction of apoptosis by a secreted lipocalin that is transcriptionally regulated by IL-3 deprivation. Science.

[44] Andrews NC (2000). Iron homeostasis: insights from genetics and animal models. Nat Rev Genet.

[45] Ratledge C, Dover LG (2000). Iron metabolism in pathogenic bacteria. Annu Rev Microbiol.

[46] Goetz DH, Holmes MA, Borregaard N, Bluhm ME, Raymond KN, Strong RK (2002). The neutrophil lipocalin NGAL is a bacteriostatic agent that interferes with siderophore-mediated iron acquisition. Mol Cell.

[47] Johnson EE, Srikanth CV, Sandgren A, Harrington L, Trebicka E, Wang L (2010). Siderocalin inhibits the intracellular replication of mycobacterium tuberculosis in macrophages. FEMS Immunol Med Microbiol.

[48] Wu H, Santoni-Rugiu E, Ralfkiaer E, Porse BT, Moser C, Hoiby N (2010). Lipocalin 2 is protective against E. coli pneumonia. Respir Res.

[49] Mowat AM, Bain CC (2011). Mucosal macrophages in intestinal homeostasis and inflammation. J Innate Immun.

[50] Smythies LE, Sellers M, Clements RH, Mosteller-Barnum M, Meng G, Benjamin WH (2005). Human intestinal macrophages display profound inflammatory anergy despite avid phagocytic and bacteriocidal activity. J Clin Invest.

[51] Nairz M, Ferring-Appel D, Casarrubea D, Sonnweber T, Viatte L, Schroll A (2015). Iron regulatory proteins mediate host resistance to Salmonella infection. Cell Host Microbe.

[52] Nairz M, Schroll A, Haschka D, Dichtl S, Sonnweber T, Theurl I (2015). Lipocalin-2 ensures host defense against Salmonella typhimurium by controlling macrophage iron homeostasis and immune response. Eur J Immunol.

[53] Bellmann-Weiler R, Schroll A, Engl S, Nairz M, Talasz H, Seifert M (2013). Neutrophil gelatinase-associated lipocalin and interleukin-10 regulate intramacrophage Chlamydia pneumoniae replication by modulating intracellular iron homeostasis. Immunobiology..

[54] Liu Z, Petersen R, Devireddy L (2013). Impaired neutrophil function in 24p3 null mice contributes to enhanced susceptibility to bacterial infections. J Immunol.

[55] Guglani L, Gopal R, Rangel-Moreno J, Junecko BF, Lin Y, Berger T (2012). Lipocalin 2 regulates inflammation during pulmonary mycobacterial infections. PLoS One.

[56] Schroll A, Eller K, Feistritzer C, Nairz M, Sonnweber T, Moser PA (2012). Lipocalin-2 ameliorates granulocyte functionality. Eur J Immunol.

[57] Cramer EP, Dahl SL, Rozell B, Knudsen KJ, Thomsen K, Moser C (2017). Lipocalin-2 from both myeloid cells and the epithelium combats Klebsiella pneumoniae lung infection in mice. Blood..

[58] Saha P, Yeoh BS, Olvera RA, Xiao X, Singh V, Awasthi D (2017). Bacterial siderophores hijack neutrophil functions. J Immunol.

[59] Fournier BM, Parkos CA (2012). The role of neutrophils during intestinal inflammation. Mucosal Immunol.

[60] Singh V, Yeoh BS, Xiao X, Kumar M, Bachman M, Borregaard N (2015). Interplay between enterobactin, myeloperoxidase and lipocalin 2 regulates *E. coli* survival in the inflamed gut. Nat Commun.

[61] Bachman MA, Lenio S, Schmidt L, Oyler JE, Weiser JN. Interaction of lipocalin 2, transferrin, and siderophores determines the replicative niche of *Klebsiella pneumoniae* during pneumonia. mBio. 2012;3(6):e00224–11. 10.1128/mBio.00224-11 PMC3509427 23169997

[62] Bachman MA, Miller VL, Weiser JN (2009). Mucosal lipocalin 2 has pro-inflammatory and iron-sequestering effects in response to bacterial enterobactin. PLoS Pathog.

[63] Bachman MA, Oyler JE, Burns SH, Caza M, Lepine F, Dozois CM (2011). Klebsiella pneumoniae yersiniabactin promotes respiratory tract infection through evasion of lipocalin 2. Infect Immun.

[64] Berger T, Togawa A, Duncan GS, Elia AJ, You-Ten A, Wakeham A (2006). Lipocalin 2-deficient mice exhibit increased sensitivity to Escherichia coli infection but not to ischemia-reperfusion injury. Proc Natl Acad Sci U S A.

[65] Roudkenar MH, Halabian R, Ghasemipour Z, Roushandeh AM, Rouhbakhsh M, Nekogoftar M (2008). Neutrophil gelatinase-associated lipocalin acts as a protective factor against H (2) O (2) toxicity. Arch Med Res.

[66] Roudkenar MH, Kuwahara Y, Baba T, Roushandeh AM, Ebishima S, Abe S (2007). Oxidative stress induced lipocalin 2 gene expression: addressing its expression under the harmful conditions. J Radiat Res.

[67] Srinivasan G, Aitken JD, Zhang B, Carvalho FA, Chassaing B, Shashidharamurthy R (2012). Lipocalin 2 deficiency dysregulates iron homeostasis and exacerbates endotoxin-induced sepsis. J Immunol.

[68] Zhao S, Wei Y, Xu D (2015). Neutrophil gelatinase-associated lipocalin attenuates injury in the rat cecal ligation and puncture model of sepsis via apoptosis inhibition. Nephrol.

[69] Clark JA, Coopersmith CM (2007). Intestinal crosstalk: a new paradigm for understanding the gut as the “motor” of critical illness. Shock..

[70] Aoyama T, Kuwahara-Arai K, Uchiyama A, Kon K, Okubo H, Yamashina S (2017). Spleen-derived lipocalin-2 in the portal vein regulates Kupffer cells activation and attenuates the development of liver fibrosis in mice. Lab Investig.

[71] Zhang J, Wu Y, Zhang Y, Leroith D, Bernlohr DA, Chen X (2008). The role of lipocalin 2 in the regulation of inflammation in adipocytes and macrophages. Mol Endocrinol.

[72] Lemaire LC, van Lanschot JB, Stoutenbeek CP, van Deventer SJ, Dankert J, Oosting H (1999). Thoracic duct in patients with multiple organ failure: no major route of bacterial translocation. Ann Surg.

[73] Adams CA, Xu DZ, Lu Q, Deitch EA (2001). Factors larger than 100 kd in post-hemorrhagic shock mesenteric lymph are toxic for endothelial cells. Surgery..

[74] Zhang Q, Raoof M, Chen Y, Sumi Y, Sursal T, Junger W (2010). Circulating mitochondrial DAMPs cause inflammatory responses to injury. Nature..

[75] Devireddy LR, Gazin C, Zhu X, Green MR (2005). A cell-surface receptor for lipocalin 24p3 selectively mediates apoptosis and iron uptake. Cell..

[76] Richardson DR (2005). 24p3 and its receptor: dawn of a new iron age?. Cell..

[77] Klausen P, Niemann CU, Cowland JB, Krabbe K, Borregaard N (2005). On mouse and man: neutrophil gelatinase associated lipocalin is not involved in apoptosis or acute response. Eur J Haematol.

[78] Johnson DE (1998). Regulation of survival pathways by IL-3 and induction of apoptosis following IL-3 withdrawal. Front Biosci.

[79] Gwira JA, Wei F, Ishibe S, Ueland JM, Barasch J, Cantley LG (2005). Expression of neutrophil gelatinase-associated lipocalin regulates epithelial morphogenesis in vitro. J Biol Chem.

[80] Jang E, Lee S, Kim JH, Kim JH, Seo JW, Lee WH (2013). Secreted protein lipocalin-2 promotes microglial M1 polarization. FASEB J.

[81] Kang SS, Ren Y, Liu CC, Kurti A, Baker KE, Bu G (2018). Lipocalin-2 protects the brain during inflammatory conditions. Mol Psychiatry.

